# Cassava postharvest physiological deterioration: a complex phenomenon involving calcium signaling, reactive oxygen species and programmed cell death

**DOI:** 10.1007/s11738-017-2382-0

**Published:** 2017-03-03

**Authors:** Astride S. M. Djabou, Luiz J. C. B. Carvalho, Qing X. Li, Nicolas Niemenak, Songbi Chen

**Affiliations:** 1 Tropical Crops Genetic Resources Institute, Chinese Academy of Tropical Agricultural Sciences/Key Laboratory of Ministry of Agriculture for Germplasm Resources Conservation and Utilization of Cassava, Hainan, China; 2 Laboratory of Plant Physiology, Department of Biological Science, Higher Teachers’ Training College, University of Yaounde I, Yaounde, Cameroon; 3 Genetic Resources and Biotechnology, Embrapa, Brazil; 4 Department of Molecular Biosciences and Bioengineering, University of Hawaii at Manoa, Honolulu, USA

**Keywords:** *Manihot esculenta*, Postharvest physiological deterioration, Calcium signaling, ROS, Programmed cell death, Crosstalk

## Abstract

Postharvest physiological deterioration (PPD) of cassava (*Manihot esculenta*) storage roots is a complex physiological and biochemical process which involve many regulatory networks linked with specific proteins modulation and signaling transduction pathways. However, it is poorly understood regarding biological regulation, and the interactions among protein groups and signals to determine PPD syndrome in cassava storage roots. This review sheds some light on the possible molecular mechanisms involved in reactive oxygen species (ROS), calcium signaling transduction, and programmed cell death (PCD) in cassava PPD syndrome. A model for predicting crosstalk among calcium signaling, ROS and PCD is suggested to fine-tune PPD syndrome. This would clues to cassava molecular breeding to alleviate the PPD effects on the shelf-life.

## Introduction

Cassava (*Manihot esculenta* Crantz) is a vegetative propagated shrub belonging to the Euphorbiaceae family. In the tropics, where it is a major staple food crop, cassava is the 4^th^ most important source of calories (Bradbury 1988). Resilience to drought and disease and tolerance to low-soil fertility enable it to grow well under a wide range of climatic conditions, where few crops could survive without costly external inputs. Despite these agronomic advantages, cassava storage roots (CSR) are far more perishable after harvest compared to other storage root and tuber crops, such as sweet potato, true yam, corn and potato. Therefore, cassava is range as a sensitive species of postharvest deterioration (An et al. 2012). The rapid deterioration of CSR significantly shortens its shelf-life for fresh consumption and impacts transportation and potential for income generation (Westby 2002; Iyer et al. 2010). This phenomenon is known as postharvest physiological deterioration (PPD). Estimated losses reach up to 8, 10, and 29% in Asia, Latin America and Caribbean, and Africa, respectively (FAO 2000). Root damage during harvest alters the equilibrium of natural physiological process of the exposed cells and subsequently their oxidative burst. Since PPD is a complex biological phenomenon, it is expected to involve early events (Buschmann et al. 2000a) as the observed dark strips of vessels due to oxidation of cell components (Apostol et al. 1989; Reilly et al. 2004) as tissue wounding reaction (Beeching et al. 2002). Later on, deterioration of cell allows microorganism growth.

This review summarized the current knowledge on oxidative events, participation of calcium signaling events and programmed cell death in association with PPD syndrome. An exploratory model was proposed for prediction of crosstalk among calcium (Ca^2+^) signaling, reactive oxygen species (ROS) production and scavenge, and apoptosis. The model could be used to predict PPD syndrome.

### Morphological and histological changes of storage root due to exposure of root cells to ambient air

**Fig. 1:**
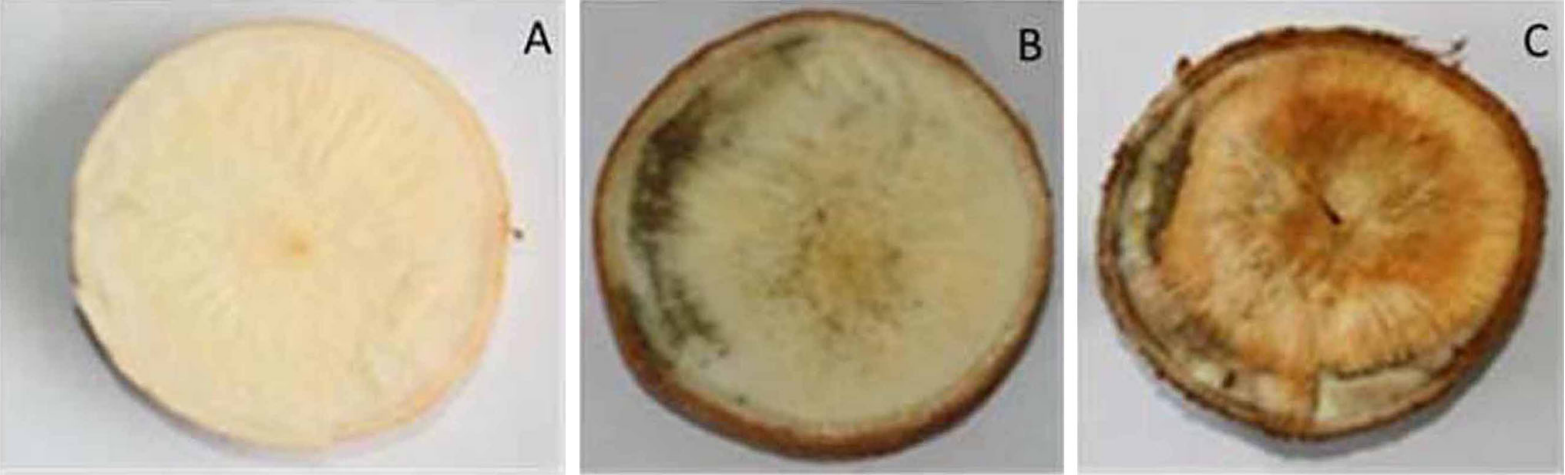
Cross sections of cassava freshly harvested storage roots exposed to air for zero (**a**), three (**b**), and ten days (**c**). Blue–black/brown discolorations recognized as a visual sign of PPD are clearly observed at 3 days. The discolorations continued until ten days following by the softening of the roots given place to development of microorganisms (secondary deterioration)

**Fig. 2:**
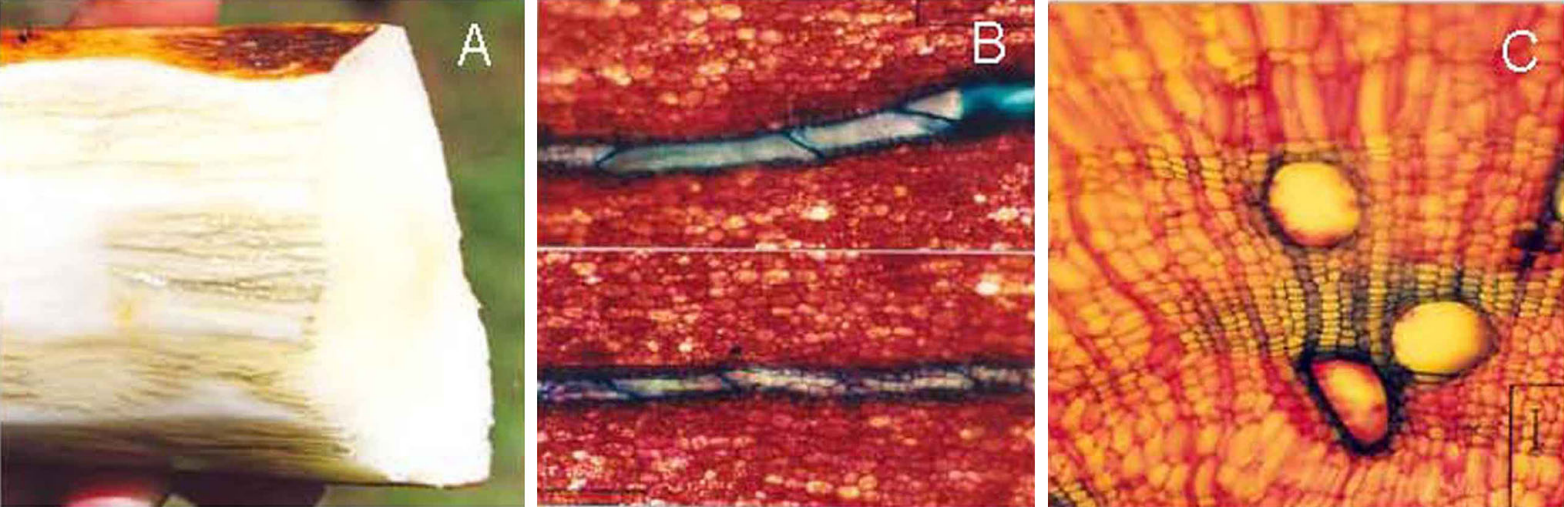
Microscopic observation of cassava storage roots undergoing PPD. **a** Dark strip formation in vessels of tissue system III resulting from oxidative process. **b** Close longitudinal cutting showing detailed vessels fully observable of tylose occlusion. **c** Close-up of tylose occlusion formation inside secondary vessels was observed

Cassava storage roots are far more perishable than other staple food crops. Subsistence and commercial utilization of cassava are affected by its short shelf-life due to a rapid postharvest physiological deterioration process (Westby et al. 2002). The duration of cassava shelf-life depends on the cultivars, harvest practices and handing, and storage conditions. However, PPD commonly occurs within 72 h after harvest and renders the root unpalatable (Buschmann et al. 2000b; Iyer et al. 2010). Formation and growth of cassava storage root are resulted from the swelling of primary roots due to the secondary growth which forms three tissue layers. The first, second and third tissue layers are composed of phellogen and phelloderm, cambium and phloem, and secondary vessels and store parenchyma cells, respectively (De Souza et al. 2006). PPD syndrome is first observed in the third tissue layer (the edible part of CSR) by visualization of dark colors changes in a cross section of a storage root (Fig. 1). This is associated with formation of the so-called dark strip of xylem vessels. Microscopic tissue sectioning observation (Fig. 2) shows that the dark strip is associated with the formation of tylose occlusions inside the secondary vessels and may be the oxidized candidate cell structure since the xylem is a dead cell. PPD considered as primary deterioration, is initiated by mechanical damage which occurs during harvesting. This is known as PPD. The visible signs are black blue to black discoloration or vascular streaking which begins at the broken or cut surfaces and subsequently spreads to the adjacent storage parenchyma and the stored starch undergoes structural changes. This primary deterioration is characterized by physiological and biochemical changes, and does not involve microorganisms (Noon and Booth 1977). Therefore, it is a biological active process and distinct from the secondary deterioration caused by microbial infection leading to softening of the root tissue (Sánchez et al. 2013; García et al. 2013). The later events of PPD syndrome involve the formation of callus from the exposed cells as healing process proceeds in addition to cell death. Difference in susceptibility to PPD amount cassava varieties has been reported (Aristizábal and Sánchez 2007; Morante et al. 2010a, b; Salcedo et al. 2010). In addition, an inversely correlation between light yellow parenchyma color of roots associated to high amount of carotenoid content and delaying of PPD was reported by Chavez et al. (2007). In fact, it is suggested that cassava wound repair can occur if the root remains attached to the plant (Plumbey and Rickard 1991; Reilly et al. 2004). Then, the problem takes place once the root is detached. Although the wound response is present, the healing process and the subsequent down-regulation of the signals are insufficient or too low (Salcedo and Siritunga 2011). It is thought that at some point during evolution cassava roots lost their efficiency in wound repair (Reilly et al. 2004). In addition, extent of PPD damage and speed of symptom development in roots was also associated to the genotypic as well as the environmental conditions increasing the complexity of the phenomenon (Reilly et al. 2004).

In the level of histology, the increase of oxidative stress caused by wounding may induce a metachromatic reaction observed by staining with toluidine blue indicating the presence of acidic polysaccharides in the cell wall and around starch granules (Uarrota et al. 2014). Acidic polysaccharides may act as reducing PPD stress. In addition, the degradation of starch granules during PPD evolution was clearly observed after staining with periodic acid Schiff (Uarrota and Maraschin 2015). Previously, Canto et al. (2013) did not observe alterations in the primary xylem after storage during PPD, suggesting that PPD process happens mainly in the peripheric region of the root without affecting the vascular cambium (primary xylem).

### Biochemical features of PPD syndrome

Several studies have been performed to investigate the biochemical feature and molecular events related to PPD syndrome (Reilly et al. 2004, 2007; Owiti et al. 2011). Changes in response to cell damages after CSR harvest included accumulation of fluorescent compounds and secondary metabolites (Buschmann et al. 2000a, b), decrease of starch content in the profit of sugar (Sánchez et al. 2013); the main soluble sugars found by high performance light chromatography were raffinose, sucrose, fructose, and glucose (Uarrota et al. 2014); increase in cell respiration and enzymatic activities including regulation of ROS synthetase (Xu et al. 2013; Zidenga et al. 2012) as well as phenylammonia-lyase. PPD has been found to be correlated with the content of β-carotene since Morante et al. (2010a, b) observed a less susceptibility to PPD for the genotypes with high level of β-carotene compared to those with less level of β-carotene. PPD development was also associated to change in gene expression where many genes get altered during the process (Huang et al. 2001; Cortés et al. 2002). Zidenga et al. (2012) suggested that mechanical damage that occurs during harvesting is initiated cyanogenesis by bringing linamarin and linamarase in contact and subsequently the release of cyanide. The cyanide (HCN) released inhibits mitochondrial respiration by inhibiting complex IV in the mitochondrial electron transfer chain. Inhibition of complex IV causes a burst of ROS production at complexes I and III. It is this oxidative burst that causes PPD. Recent studies about metabolome analyses showed increases in carotenoids, flavonoids, anthocyanins, phen-olics, reactive scavenging species, and enzymes (superoxide dismutase family, hydrogen peroxide, and catalase) under PPD (Uarrota et al. 2014). In the same study, a positive correlation was observed between PPD and antocyanins and flavonoids while a negative correlation was observed with phenolics compounds and carotenoids. Several proteins were up- or down-regulated during the process (Owiti et al. 2011). Plant responds to various stresses such as pathogen attacks, harsh growing conditions, wounding by inducing the expression of a large number of genes that encode diverse proteins. The response of plant tissues to wounding has been studied for a very long time and more recently it has been demonstrated that several genes are wound-inducible. The proteomic approach is a very powerful tool to study the proteins patterns that result from differential gene expression as well as from post-translational modifications (Gray and Heath 2005; Lee et al. 2007; Timperio et al. 2008). Proteome profile of CSR at harvest and during PPD onset revealed 300 proteins showing significant abundance regulation during PPD (Vanderschuren et al. 2014). The identified proteins were mostly associated with oxidative stress, phenylpropanoid biosynthesis (including scopoletin), the glutathione cycle, fatty acid-oxidation, folate transformation, and the sulfate reduction II pathway in which glutathione peroxidase was identified as a possible candidate for reducing PPD. All the information clearly showed that PPD is an active and complex phenomenon involving the possible interaction/crosstalk between the pathways.

### The role of ROS in plants

Plants and other living organisms in the oxidizing environment constantly produce ROS from chloroplasts, mitochondria, peroxisomes and other subcellular organelles because of biological metabolic processes such as photosynthesis and respiration. Overproduction of ROS is triggered by a pathogen attack and stress exerted by environmental conditions. ROS or active oxygen species (AOS) or reactive oxygen intermediates (ROI) include the superoxide radical (O_2_^−^), hydroxyl radical (OH·), hydroperoxyl radical (HO_2_^·^), hydrogen peroxide (H_2_O_2_), alkoxy radical (RO·), peroxy radical (ROO·) and singlet oxygen (^1^O_2_) (Vellosillo et al. 2010). Plants have either enzymatic or non-enzymatic defense systems to scavenge ROS toxicity and protect against oxidative damage (Vranova and Inze 2002). The enzymatic scavenging system includes superoxide dismutase (SOD), ascorbate peroxidase (APX), catalase (CAT), glutathione peroxidase and glutathione reductase while the non-enzymatic scavenging system includes ascorbic acid (AsA), glutathione and proline. In response to environmental stresses, plants produce increased levels of ROS and SOD which provides the first line of defense is, thus, important in plant stress tolerance (Apel and Hirt 2004). The increase of glutathione reductase activity confers stress tolerance and has the ability to alter the redox state of important components of the electron transport chain (Foyer and Noctor 2011). The organism equilibrium is determined by the ROS homeostasis, including ROS producing in combination with its scavenging. Contact with invading microbe as well as wounding in many plants elevates plant plasma membrane-bound NADPH activity, leading to the rapid accumulation of ROS including hydrogen peroxide (H_2_O_2_), which diffuses into cells and activates defenses (Apel and Hirt 2004). Increasing evidence indicates that ROS has a dual role as cytotoxic damage, and as a signaling molecule involved in the regulation of response during pathogen attack or various physiological processes (Foyer and Noctor 2005; Mittler et al. 2004; Corpas et al. 2015). The accumulation of ROS in the site of infection during hypersensitive response may contribute to limit the spread of the pathogens or induce signals for establishment of further defense (Mur et al. 2008). Feed-back, or feed-forward interaction between ROS and many hormones such as ethylene, jasmonic acid, abscisic acid, gibberellic acid and salicylic acid in response to biotic and abiotic stresses has also been presented (Mittler et al. 2004; Mur et al. 2008). Since ROS is ideally suited to act as signaling molecules because of its small size and ability to diffuse over short distances (Mittler et al. 2004; Pei et al. 2000; Overmyer et al. 2000), it is not surprisingly that numerous researchers paid close attention to the oxidative burst in damage cassava storage roots.

### Features of ROS associated to PPD syndrome

Cassava storage root is inevitable predisposed to cell damage due to mechanical injury caused by harvest. Therefore, constitutive defense mechanisms are activated upon harvest as in intact plant subjected to abiotic stress. The response of cell damage produces ROS including superoxide anion and hydrogen peroxide with both local and systemic action (Apel and Hirt 2004). In the case of CSR, studies have been focused on the ROS production and their scavenging induced by cell damage during PPD syndrome (Xu et al. 2013; Zidenga et al. 2012). A burst of superoxide anion and the increased activities of ROS scavenging enzymes such as superoxide dismutase and catalase were observed after oxidative burst caused by harvest damage (Iyer et al. 2010; Reilly et al. 2007). The shortage of scavenge of the overproduced ROS results in accelerate PPD. Previously, Zidenga et al. (2012) suggested that the cyanide produce immediately when cassava is mechanically damaged may trigger the oxidative burst responsible of PPD onset. In fact cassava produces potentially toxic levels of cyanogenic glucosides which break down to release cyanide following cellular disruption and release cyanogens from the vacuole (Siritunga and Sayre 2003; Siritunga et al. 2004). Therefore, increasing the production of ROS scavenges is presented as a key regulator of PPD. The induction of the overexpression of mitochondrial alternative oxidase (AOX) in transgenic cassava was followed by extending of the shelf-life of storage root (SR) for two weeks (Zidenga et al. 2012). Parallel experiments showed that the co-overexpression of Me/Cu/Zn-SOD and MeCAT1 induced in transgenic cassava could also enhance scavenging ROS in CSR after tissue damage (Xu et al. 2013). In addition, the regulation of the activity of glutathione-associated enzymes, including glutathione reductases, glutaredoxins, and glutathione S-transferases have been reported as potential modulator for the onset of PPD (Vanderschuren et al. 2014). Recently, increase in APX, GPX, AsA, and CAT subsequently detoxify the hydrogen peroxide was recently reported in cassava roots during PPD (Uarrota and Maraschin 2015). Taken together these observations strongly support the implication of ROS during PPD process and present many pathways which may trigger their production.

### Features of calcium signaling associated to PPD syndrome

Plant cells could trigger their elaborate defense systems while perceiving signal messenger coming from their environment, and eventually produce proper physiological responses. In plants, calcium ion (Ca^2+^) is a ubiquitous second messenger molecule coupling with physiological response to external and developmental signals (Reddy and Reddy 2004). It plays a key role in the integrity of the cell wall and the membrane systems and acts as an intracellular regulator in many aspects of plant growth and development including stress responses (White and Breadly 2003). Changes in cytosolic free Ca^2+^ concentration were observed during transduction of abiotic stimuli including high light, low and high temperature, hyperosmotic and oxidative stresses and also in the biotic stimuli including fungal elicitors and nodulation factors (Rudd and Franklin-Tong 2001). These Ca^2+^ signatures are recognized by several types of Ca^2+^-sensor proteins. Ca^2+^-binding sensory proteins include calmodulins (CaMs), calmodulin-like proteins, calcineurin B-like proteins (CBL), and Ca^2+^-dependent protein kinases (CDPKs) (Sanders et al. 2002; Snedden and Fromm 2003).

There is an ample evidence to show the involvement of Ca^2+^ signaling in abiotic stress responses. Intracellular changes of Ca^2+^ levels were reported as the first response to diverse abiotic signals and biotic stresses (Beneloujaephajri et al. 2013; Ranty et al. 2006). Numerous data supported that Ca^2+^ and its sensor proteins, CaM, CDPKs and CBLs and downstream elements played an important role in plant adaptation to abiotic stress. Several studies indicated Ca^2+^-CaM complex triggered the activation of target proteins to produce cellular physiological responses (Bouché et al. 2005; Nookaraju et al. 2011). An example was found in tobacco stressed by wounding, in which three CaM isoforms at different Ca^2+^ concentrations activate the target enzymes NO synthase and NAD kinase (Karita et al. 2004). Similar effect of Ca^2+^ on NO or NAD kinase during PPD cannot be ruled out.

In the case of cell injuries such as the one occurring in PPD syndrome of CSR, a significant up-regulation of CaM observed at the early events of PPD (Owiti et al. 2011) can be associated with a rapid increase in Ca^2+^ which resulted in the oxidative burst as observed in *Arabidopsis thaliana* (Kaplan et al. 2006). This phenomenon was similar to the expression of heat shock proteins under heat stress attributed to the accumulation of CaM in plants (Liu et al. 2003; Zhang et al. 2009). Ca^2+^ seems to be involved in signaling transduction to trigger the activation of programed cell death (PCD) (Zhang et al. 2009; Hoeberichts and Woltering 2002; Levine et al. 1996). It was suggested that CDPKs, in response to elevated cytosolic Ca^2+^ levels, could induce NADPH oxidase activity which is one of the key points of an oxidative burst and PCD process (Hoeberichts and Woltering 2002). In roots of *Arabidopsis thaliana*, mechanical stimulation triggered the rapid and transient increase of cytoplasmic Ca^2+^ concentration; this mechanical stimulation likewise elicits apoplastic ROS production with the same kinetics (Monshausen et al. 2009).

### Programmed cell death features in plants

Programmed cell death (PCD) is an active and genetically controlled process aimed at eliminating redundant or harmful cells from healthy tissues during the life cycle of the multicellular organism. It is a highly regulated cellular suicide process and essential for growth and survival in eukaryotes. The biochemical and morphological hallmarks of PCD recorded to be common to plants and animals are compaction and shrinkage of the cytoplasm and nucleus, DNA and nuclear fragmentation into large (50–300 kb) and subsequently small nucleosomal fragment (200 bp, DNA laddering) and calcium influx (De Jong et al. 2000; Wang et al. 1996). PCD was reported in many biological processes in plants including embryogenesis, flower petal senescence and vegetative development such as xylogenesis, and parenchyma formation. Various stress conditions such as cold, nutrient deprivation, salts or d-mannose stresses, pathogen or pathogen toxin (Wang et al. 1996) have been found to induced DNA laddering and subsequently death in plants. PCD is also a process involving new protein synthesis and distinct from necrosis, cell death caused by extrinsic factors, and independent of specific genetic control and cellular activities (Van Breusegem and Dat 2006).

The core component of the apoptotic machinery found in animals is a proteolytic cascade involving a family of cysteine proteases named caspases, which specifically cleave at aspartic acid residues of their substrates (Shi 2002; Zhang et al. 2009). This cleavage may be involved in the detachment of the dying cell making it easy to ingest (Shi 2002). Even if no homologs of animal’s caspases have been identified in plants, accumulated evidence in the recent years suggests caspases-like activity playing a pivotal role in plant PCD (He et al. 2008; Woltering et al. 2002). The expression of caspase-3-like has been detected from barley embryonic suspension cells and TMV-infected tobacco leaves (Lam and Zhang 2012). Parallel experiments were performed to show the cleavage of Poly (ADP Ribose) polymerase, a specific substrate of caspase-3 during the apoptotic process in animal by extracting from fungus-infected cowpea. Comparing to the animal apoptotic pathway, this degradation was dependent on the release of cytochrome c into the cytosol and could be inhibited by specifics caspase 3 inhibitors. Using the synthetic fluorogenic caspase-1 substrate (Ac-DEVD-AMC), caspase-like activity was also detected during UV- or heat shock-induced apoptosis of plant cells, and this could be inhibited by caspase-3 inhibitors but not by caspase-unrelated protease (Vacca et al. 2006). All of these experiments evidenced that the functional caspase-like proteolytic activity was detected and its functional involvement in plant cells undergoing PCD.

Recently, the metacaspase, a homolog of caspase, implicated in plant PCD was also detected (Koonin and Aravind 2002; Uren et al. 2000). Even if it was unable to cleave caspase substrates, it was found to be involved in PCD in *Arabidopsis* and yeast (He et al. 2008; Madeo et al. 2002). The level of cell death was increased in transgenic plant by overexpressing some *Arabidopsis* metacaspases (AtMC4, AtMC5) upon treatment with ROS inducing agents and the loss of those genes resulted in a decrease or delay of cell death (Lam and Zhang 2012). PCD following oxidative burst has been demonstrated in many plants (Woltering et al. 2002; Vacca et al. 2006; Danon et al. 2000). Several calcium-binding proteins were induced in response to the stresses. CDPKs, in response to elevated cytosolic Ca^2+^ level, can induce NADPH oxidase, which catalyzes the production of ROS such as superoxides (Sagi and Fluhr 2006). Even if the oxidative burst induced by wound in cassava storage root after harvest has been studied, the linkages between oxidative burst and PCD are poorly understood. In fact, high concentrations of ROS are highly harmful to organisms, and when the symptoms persist, irreversible damage may occur to the cells, resulting in loss of physiological capacity and eventual cell death.

Regarding cassava storage root PPD, PCD process was marked by the down-regulation of cysteine protease which may enhance protease activity leading to PCD as well as the down regulation of many peptides of the phospholipase D α-group of the enzymes during the early and late PPD times points were also presented (Owiti et al. 2011). However, further studies need to be carried out to describe how this pathway works in a PPD process. Biochemical detection of caspase-3-like activity and its inhibitors in plant PCD can be useful to elucidate the mechanism of PPD. Apart from this further functional characterization, understanding of the mechanism on which this pathway interacts with other pathways such as Ca^2+^ and ROS like mentioned in others plants during stress can increase the knowledge about the PPD process in cassava.

### Crosstalk among Ca^2+^ signaling, ROS and PCD integration network in cassava to fine-tune PPD syndrome

In cassava, the events that trigger the production of ROS relevant to PPD under abiotic and biotic stresses are poorly understood. Information is rising about the implication of Ca^2+^ in the release of ROS in wound-induced resistance in plant (Beneloujaephajri et al. 2013; Sagi and Fluhr 2006; Bargmann and Munnik 2006). In response to wound induced by *Botrytis cinerea*, it was found that non-wounded leaves of *Arabidopsis thaliana* treated with Ca^2+^ inhibitors were more susceptible to pathogen, suggesting the importance of Ca^2+^ in the induction of basic resistance (Beneloujaephajri et al. 2013). Interestingly, a co-localization of the changes in Ca^2+^ and a burst of ROS were observed after pathogenic or environmental stresses were exerted (Monshausen et al. 2009; Ranf et al. 2011). The transient changes in Ca^2+^ concentration were detected for a few seconds after wounding, followed by the increase of ROS concentration in *A. thaliana* leaves after wounding (Beneloujaephajri et al. 2013). Indeed, in plants a positive feedback mechanism involving NADPH oxidase, ROS and Ca^2+^ was (Sagi and Fluhr 2006). Various Ca^2+^ binding proteins such as CDPKs in response to elevated cytosolic Ca^2+^ levels could induce NADPH oxidase activity, leading to the increase of ROS under stresses (Sagi and Fluhr 2006), and potentially trigger other downstream responses such as apoptosis. CDPK6, a Ca^2+^ related protein of *A. thaliana*, was reported to be involved in the regulation of ROS (Boudsocq and Sheen 2010). An ortholog of this protein found in potato was involved in the phosphorylation of the membrane-bound NADPH oxido-reductase RBOH-D to stimulate its activity for ROS production in response to pathogen attacks (Kobayashi et al. 2007). However, ROS production from the initial Ca^2+^-dependent activation can subsequently trigger a larger Ca^2+^ influx (Pei et al. 2000; Kadota et al. 2004). Thus, it is possible that Ca^2+^ cytosolic elevation is crucial for ROS accumulation, which in turn contributes to Ca^2+^ signaling in positive feedback loop (Sagi and Fluhr 2006; Bargmann and Munnik 2006). The crosstalk between the second messengers Ca^2+^ and ROS is now recognized in the modulation of the activity of specific proteins that act at the nuclear level to control the expression of determinate defense genes. In plants, Ca^2+^ fluxes were also recognized as an important signaling mediator of the activation of PCD and NADPH oxidase complexes may be stimulated by caspase (-like) activity (Hoeberichts and Woltering 2002). They may trigger the reduction of oxygen to ^·^O_2_^−^ followed by dismutation of ^·^O_2_^−^ to H_2_O_2_. Subsequently, caspase inhibitors can act to prevent cell death and the preceding accumulation of ROS. Increasing evidence indicated that an interaction may exist between elevated cytosolic Ca^2+^, accumulation of ROS and subsequent cell damage. Zidenga et al. (2012) showed that the accumulation of ROS during PPD can be the consequence of cyanide released during cyanogenesis and did not observe a substantial reduction of ROS production with diphenyl iodonium chloride an inhibitor of membrane NADPH oxidase. The up-regulation of CaM one of the most important calcium sensors proteins coupled to the increase of cysteine protease during early PPD suggest the implication of others pathways on PPD process recognize as an active process. Growth regulators can interact in coordination under various stress conditions in order to control the downstream stress response or with others pathways to fine-tune the defenses (Verma et al. 2016). The response of plants imposed by abiotic stress such as wound in cassava roots could be mainly controlled by growth regulators even if it remains poorly understood. Taking all information together PPD induced in CSR seems to reflect an integrative crosstalk between signaling molecules, including Ca^2+^-CaM/CDPK, ROS, hormones such as jasmonic acid, salicylic acid, ethylene, gibberellin acid, cysteine protease and still unknown members of PCD pathway.

**Fig. 3:**
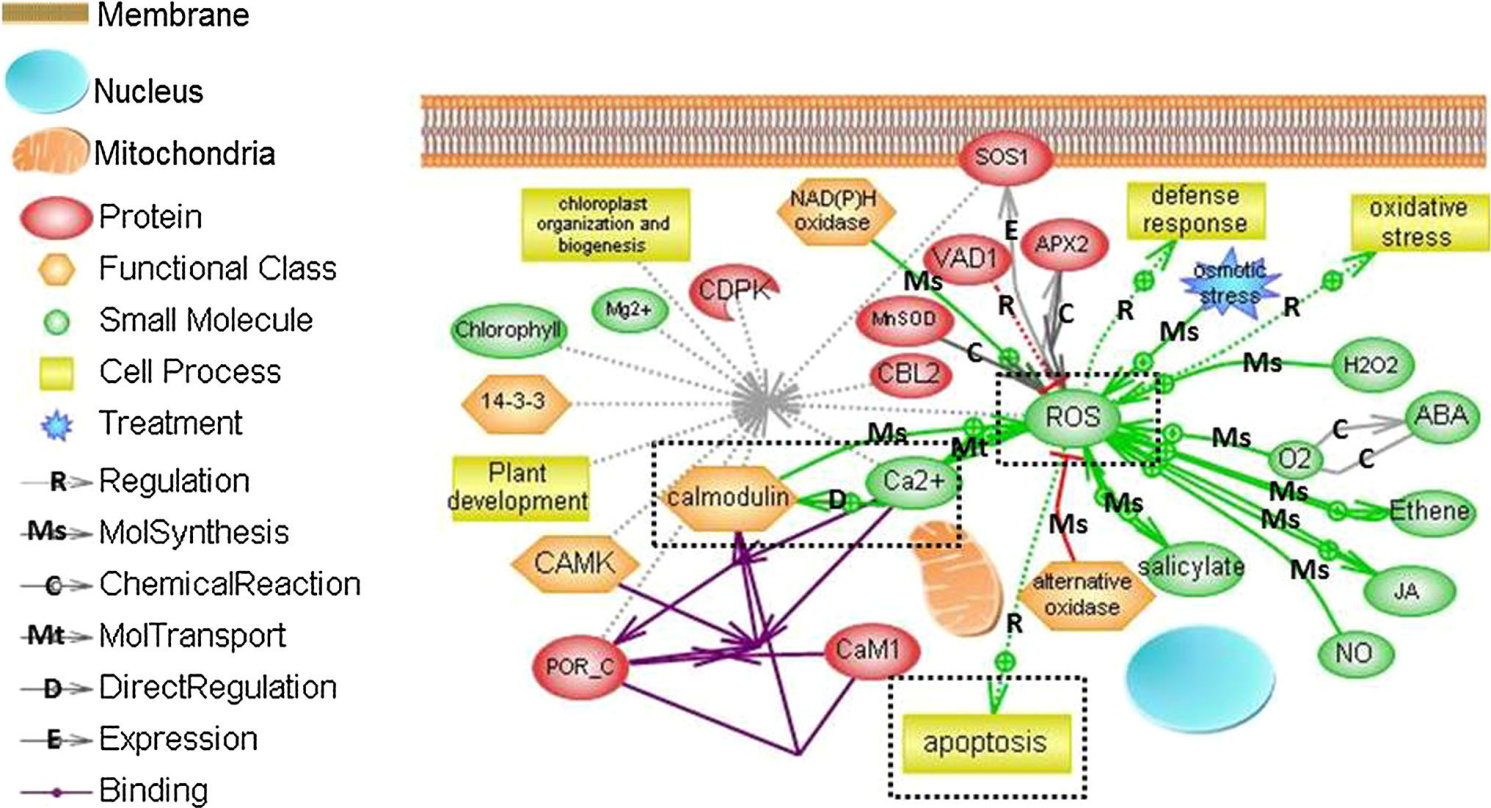
Putative crosstalk among Ca^2+^, ROS and apoptosis in cassava storage roots during PPD. The network was generated with Pathway Studio software default. Regulation is marked as an *arrow with R*, MolSynthesis as an *arrow with Ms*, Chemical Reaction as an *arrow with C*, MolTransport as an *arrow with Mt*, Direct Regulation as an *arrow with D*, Expression as an *arrow with E* and Binding as an *arrow without any marks*

Based on the data described above, we would propose a mechanism of PPD in cassava. All impact factors associated with PPD were used to generate a biological interaction network using Pathway Studio. This network includes cell process, functional class, protein, small molecule and osmotic stress treatment. The relationship among chemical reaction, expression, regulation, and binding was established (Fig. 3) responding to defense response and oxidative stresses caused from wound damage and microbial infection. Mechanical damage causes by wound induces oxidative burst and stimulates Ca^2+^ influx. This flux is sensed by Ca^2+^ binding proteins such as CaM which has several Ca^2+^-dependent in vitro activities. It is involved in regulating various cellular and biochemical processes, such as PPD. Ca^2+^ signaling was essential for activating the NO and ROS production induced by mechanical damage. Therefore, Ca^2+^-CaM complex is a key center to regulate ROS homeostasis. Other key points are ROS and apoptosis. ROS are responsible for mediating cellular defense responses in cassava. The production of ROS, mediated through NADPH oxidase, increases under stress conditions such as wound damage, causing oxidative burst and impairment of normal metabolism. ROS are also key elements in cassava PCD which is essential for microbial infection. In the model, a negative control on cell death-dependent ROS accumulation, promoted by SA and ethylene is limited by jasmonic acid (Fig. 3). In addition, CAT and SOD can interfere with hormones to scavenge the flux of ROS produced restore the equilibrium and PPD can be delayed. In contrary, the increasing production of ROS can trigger both a second peak of Ca^2+^_cyt_ which interfere with mitochondrion and the production of MAPK. MAPK can act with heat shock proteins and others cell death pathway inducing cell wall degradation and subsequently PPD and PCD. All together, we consider PPD in cassava as a complex process in which the crosstalk among Ca^2+^ signaling, ROS and PCD is integrated to fine-tune.

## Conclusion

Cassava storage roots is an essential part of the daily diet for millions people and income generation. However, its potential as food and industrial crop is still limited by PPD. The study of PPD mechanism response is important in increasing our molecular knowledge and for their potential in developing an effective approach to control PPD losses. The recent data revealed the implication of Ca^2+^-CaM, ROS and PCD pathways in response to PPD. The crosstalk between these pathways during PPD process is proposed in this review. However, more functional studies are needed to better understand when, where and how the proteins involved in these pathways talk or interact to fine-tune the PPD response. Progress in proteomic, cell image technology and molecular genetic analysis will be helpful to drive future research and provide a worthwhile approach to control PPD in cassava storage roots.

### **Author contribution statement**

SC made the major contributions to this study in the conception, design, drafting part of manuscript, and final revision. ASMD contributed to part of the conception and design, and drafting manuscript. LJCBC worked at Fig. 2 and revision of manuscript. QXL contributed to part of conception, and critical revision of manuscript. NN worked at part of design and critical revision of manuscript. All authors read and approved the final manuscript.
